# Vitamin D: A key player in COVID-19 immunity and lessons from the pandemic to combat immune-evasive variants

**DOI:** 10.1007/s10787-024-01578-w

**Published:** 2024-10-16

**Authors:** Hussein Sabit, Shaimaa Abdel-Ghany, Mahmoud S. Abdallah, Osama Abul-Maaty, Ahmed I. Khoder, Nabil A. Shoman, Mohamed Sameh Farrag, Pavel Martasek, Ayman M. Noreddin, Mahmoud Nazih

**Affiliations:** 1 Department of Medical Biotechnology, College of Biotechnology, Misr University for Science and Technology, P. O. Box 77, Giza, Egypt; 2 Department of Environmental Biotechnology, College of Biotechnology, Misr University for Science and Technology, P. O. Box 77, Giza, Egypt; 3 Department of Clinical Pharmacy, Faculty of Pharmacy, University of Sadat City (USC), Sadat City, 32897 Egypt; 4 Department of PharmD, Faculty of Pharmacy, Jadara University, Irbid, 21110 Jordan; 5 Faculty of Science, Mansoura University, Mansoura, Egypt; 6 Scientific Office, Egyptian Society of Pharmacogenomics and Personalized Medicine (ESPM), Cairo, Egypt; 7 Molecular Biology Department, Genetic Engineering and Biotechnology Research Institute, University of Sadat City, Sadat City, Egypt; 8 Pharmacology and Toxicology Department, Faculty of Pharmacy, Menoufia University, Shebin El-Koum, Egypt; 9 Department of Pharmaceutics and Pharmaceutical Technology, Faculty of Pharmacy, Ahram Canadian University, Giza, Egypt; 10 Department of Paediatrics and Inherited Metabolic Disorders, First Faculty of Medicine, Charles University and General University Hospital in Prague Ke Karlovu 2, 128 08 Praha 2, Czech Republic; 11 Department of Clinical Pharmacy, Faculty of Pharmacy, Ahram Canadian University (ACU), 6th of October City, Giza, 12566 Egypt; 12 Department of Internal Medicine, School of Medicine, University of California-Irvine, Irvine, CA USA; 13 Al Ryada University for Science and Technology (RST), ElMehwar ElMarkazy-2, Cairo - Alex desert RD K92, Sadat City, 16504 Egypt

**Keywords:** SARS-CoV-2, COVID-19, COVID-19 variants, Vitamin D, Vitamin D deficiency

## Abstract

As of the 7^th^ of July 2024, 775,754,322 confirmed cases of COVID-19, including 7,053,902 deaths worldwide, had been reported to the WHO (World Health Organization). Nevertheless, untill the 15^th^ of July 2024, a total of 13,578,710,228 vaccine doses had been administered, with almost no country spared from COVID-19 attacks. The pathophysiology of this virus is complicated, and several symptoms require a deep understanding of the actual mechanisms. It is unclear why some patients develop severe symptoms while others do not, although literature suggests a role for vitamin D. Vitamin D plays a crucial role in the infection or in ameliorating the severity of symptoms. The mechanism of action of vitamin D and vitamin D deficiency (VDD) is well understood. VDD is associated with increased hospitalization of severely ill patients and increased levels of COVID-19-caused mortality. Recent studies suggest that vitamin D levels and genetic variations in the vitamin D receptor (VDR) gene significantly impact the severity and outcomes of COVID-19, especially in the infections caused by Delta and Omicron variants. Furthermore, VDD causes immune system dysregulation upon infection with SARS-CoV-2, indicating that vitamin D sufficiency is crucial in fighting against COVID-19 infection. The therapeutic effect of vitamin D raises interest in its potential role as a prophylactic and treatment adjunct. We evaluate the immunomodulatory effects of vitamin D and its ability to enhance the efficacy of new antiviral drugs like molnupiravir and paxlovid against SARS-CoV-2. This review discusses the role of vitamin D sufficiency and VDD in COVID-19 initiation and progression, emphasizing the molecular mechanisms by which vitamin D exerts its actions as a proactive step for the next pandemic. However, there is still no clear evidence of vitamin D’s impact on prevention and treatment, leading to contradictory findings. Therefore, large-scale randomized trials are required to reach a definitive conclusion. A bibliometric analysis of publications related to vitamin D, immunity, and COVID-19 revealed a significant increase in research activity in this area, particularly in 2020–2024, underscoring the growing recognition of vitamin D’s potential role in the context of the pandemic.

## Introduction

Coronaviruses are of four types: α, β, γ, and δ. SARS-CoV, MERS-CoV, and SARS-CoV-2 are β-coronaviruses. Genetic analysis shows that SARS-CoV-2 shares an 89.1% nucleotide sequence similarity with SARS-CoV, indicating a closer relationship than MERS-CoV (El-Hddad et al. 2024). SARS-CoV-2 is the virus that leads to COVID-19 disease. COVID-19 is a contagious disease presented by several clinical symptoms ranging from mild to potentially critical illness. Clinical manifestations include dry cough, shortness of breath, loss of smell and taste, bilateral viral pneumonia, muscle pain, fever, headache, acute respiratory distress syndromes, respiratory failure, and cytokine release syndrome (Meyerowitz et al. 2024). These symptoms range from flu-like to death (Hussain et al. 2020). COVID-19 was declared a global pandemic due to its high contagiousness, as well as worldwide morbidities and mortalities (Khan et al. 2020; Rehman et al. 2020), and no country has been spared from its onslaught. COVID-19 infections initiate an acute inflammatory response and oxidative stress, leading to acute respiratory distress syndrome (ARDS) and death in the worst case (Las et al. 2020). Nearly 80 million cases have been affected by this virus, though the next pandemic will be less virulent due to the preparation actions employed; however, lessons regarding VDD should be learned.

Even though it is not widely practiced, vitamin D has been proposed as one of the potential lines in preventing severe manifestations, bad prognosis, complications, and deaths from the disease (Brenner and Vitamin 2021). Vitamin D can be obtained from skin synthesis after solar UV-B exposure, diet, or supplementation (Zittermann et al. 2006). Vitamin D deficiency has been reported to be associated with respiratory tract infection (Barassi et al. 2021; Demir et al. 2021), although it is preventable by supplementation (Griffin et al. 2020). It is unclear why some patients develop severe symptoms upon contracting the infection of SARS-CoV-2 while others do not. Nonetheless, many reports have suggested vitamin D as a key factor in reducing the risk of either infection or developing severe symptoms (D’Avolio et al. 2020). Recent work has suggested a possible role of VDD in the dysregulation of cytokine production and inflammatory response as key complications of COVID-19 infection. Furthermore, vitamin D can regulate the expression of several genes in the immune cells because of its nature as a steroid hormone (Griffin et al. 2020).

This review summarizes the current knowledge about the role of VDD in the pathophysiology of COVID-19, emphasizing the molecular mechanism by which vitamin D exerts its functions. This would help in the awareness strategies to protect people from developing severe manifestations and complications from the viral infection in case we face an upcoming pandemic. Molnupiravir and Paxlovid have also recently gained attention due to their effectiveness in treating COVID-19. This article delves into the relationship between these drugs and vitamin D, a crucial nutrient with known immune-boosting properties. In addition, we will summarize current knowledge on the multifaceted role of vitamin D in preventing and treating COVID-19 and its variants.

## Vitamin D structure

**Fig. 1:**
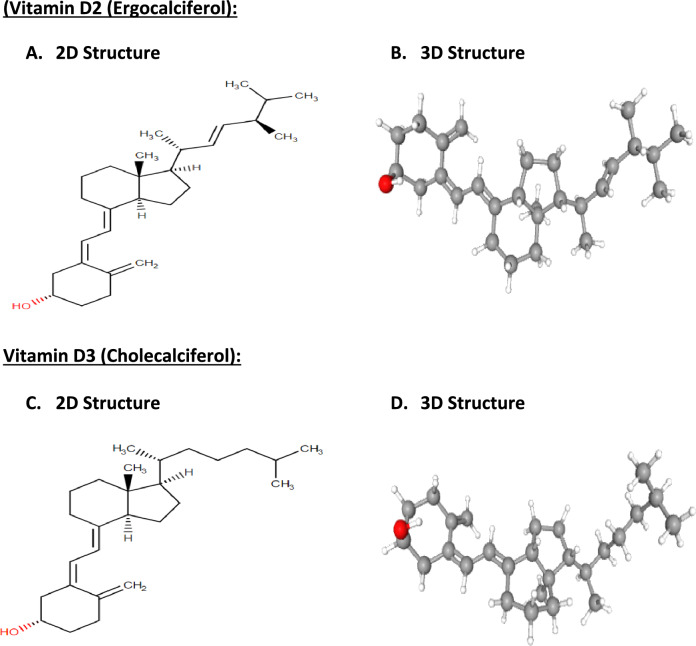
Overview of vitamin D structure and its structure–activity relationships (SARs). The most biologically active forms of vitamin D are vitamin D2 (ergocalciferol) (**A**, **B**) and vitamin D3 (cholecalciferol). **C**, **D**. Ergocalciferol has a chemical structure characterized by a steroidal backbone. Its 3D structure includes a tetracyclic ring system with a side chain. Vitamin D3 (cholecalciferol) is structurally similar to vitamin D2, but differs in the side chain. It also contains a steroidal backbone with a tetracyclic ring system

Vitamin D refers to a group of fat-soluble secosteroid hormones that play a vital role in regulating calcium and phosphate metabolism, bone health, and immune function (Christakos et al. 2011; Bishop et al. 2021). Vitamin D3 (cholecalciferol) is the predominant form of vitamin D in humans and animals, characterized by a steroid backbone with four fused rings. Vitamin D2 (ergocalciferol), another variant of vitamin D, shares a tetracyclic ring system but differs in its side chain composition (Jäpelt and Jakobsen 2013; Norman 2008). In comparison, cholecalciferol possesses a characteristic secosteroid structure with a broken ring that allows conformational flexibility important for binding to the vitamin D receptor (Molnár et al. 2011; Norman 2006). The 2D and 3D structures of vitamin D2 and D3 are presented in Fig. 1.

Structure–activity relationship (SAR) analysis examines how modifications to the chemical structure of a compound influence its biological activity. For vitamin D, understanding SARs is key to elucidating its functions and enabling analog development for therapeutic applications (Mizwicki and Norman 2009). Inactive vitamin D undergoes activation reactions, becoming potent ligands for the target tissues’ vitamin D receptor (VDR) (Jones et al. 2012). Binding triggers effects on calcium/phosphate homeostasis, bone health, immunity, and cell growth (Haussler et al. 1997). Researchers have synthesized analogs with tailored side chain and ring alterations to selectively enhance the desired actions or reduce adverse effects (Etten and Mathieu 2005; Nagpal et al. 2005). This has enabled the creation of synthetic derivatives like calcitriol to treat osteoporosis (Molnár et al. 2006).

The vitamin D scaffold, containing a broken secosteroid ring, flexible side chain, and cis-triene motif, presents specific binding groups to VDR in an optimal orientation (Norman 2008; Zhu et al. 2013). The A-ring 3-hydroxyl enables receptor binding, while the adjustable side chain allows fitting into VDR’s ligand pocket (Molnár et al. 2011; Sutton and MacDonald 2003). The cis-triene component contributes to shape and binding affinity (Norman 2008). Even minor modifications to these structures significantly impact vitamin D bioactivity (Mizwicki and Norman 2009; Bishop et al. 2021; Carlberg and Campbell 2013). Further insights into SARs will aid in harnessing the full therapeutic potential of vitamin D analogs.

## Vitamin D sources and functions

Vitamin D (cholecalciferol, C_27_ H_44_O) is an essential hormone for human health. It is a fat-soluble vitamin that can be synthesized in the skin after exposure to UV or from dietary sources. Vitamin D is synthesized in the skin from 7-dehydrocholesterol (Holick et al. 1987; Crissey et al. 2003). 7-Dehydrocholesterol reacts with UVB at wavelengths between 290 and 315 nm present in sunlight. The epidermis of young and adult individuals is the major site of vitamin D synthesis, accounting for more than 80% of the total vitamin D synthesized in the skin (MacLaughlin and Holick 1985). Vitamin D is a prohormone that undergoes conversion to the active form, 1,25-dihydroxy vitamin D [1,25(OH)(2)D]. The active form of vitamin D interacts with its receptor, vitamin D receptor (VDR), to modulate the expression level of several genes associated with the biological responses (Dusso et al. 2005; Khazai et al. 2008). Recent reports indicated that maintaining optimal vitamin D at optimal levels is crucial in reducing the risk of several chronic (Matyjaszek-Matuszek et al. 2015) and infectious (Mercola et al. 2020; Kumar et al. 2021) diseases.

Vitamin D increases calcium levels to ensure a sufficient blood level (Wasserman 2004; Christakos et al. 2011). It also promotes calcium absorption within the small intestines (Christakos et al. 2011) and reabsorption in renal tubules, thereby decreasing calcium excretion (Veldurthy et al. 2016). Moreover, vitamin D increases the absorption of magnesium (Hardwick et al. 1991; Hodgkinson et al. 1979) and phosphorus (Welch et al. 2017) within the intestine.

**Fig. 2:**
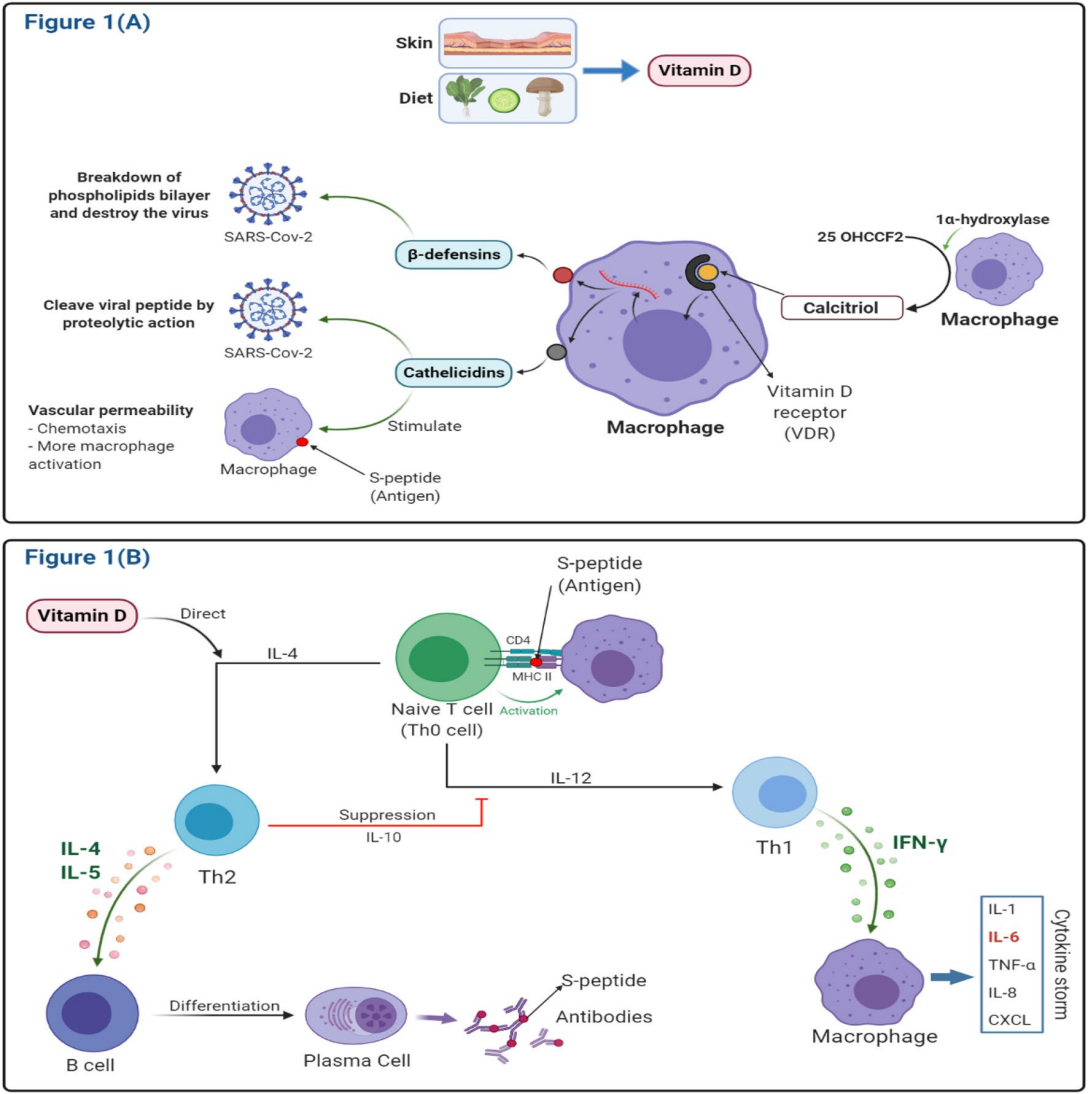
The general role of vitamin D in immune system modulation. **A** The synthesis of vitamin D by either diet or exposure to UV-B. The provitamin is converted in macrophage into the active form of vitamin D via 1α-hydroxylase. The active form of vitamin D then induces the expression of cathelicidins and β-defensins from macrophages that help fight the SARS-CoV-2 virus. Finally, macrophages represent the S-peptide of the virus to naïve T cells. **B** Naïve T cells are matured to Th2 in the presence of vitamin D and finally to plasma cells that produce specific antibodies against S-peptide. In the case of VDD, naïve T cells are matured to Th1 (the bad pathway), which produces a cytokine storm

When obtained from food or synthesis in the skin, vitamin D undergoes two types of conversions within the human body (Fig. 2a, 1b). In the liver, with the aid of 25-hydroxylase, cholecalciferol undergoes conversion into 25-hydroxy cholecalciferol (25(OH)vitamin D). This compound circulates to the kidney, where it undergoes the second conversion with the aid of 1α-hydroxylase into 1,25-dihydroxycholecalciferol (*aka* calcitriol, the active form of vitamin D) (Bikle 2014). As a steroid hormone, this active form binds to vitamin D receptors (VDRs) (Webb 2006), which are located in the nuclei of target cells (Malloy et al. 2009), whereby the expression and repression of the different genes take place (Pike and Meyer 2010; Valdivielso 2009).

**Fig. 3:**
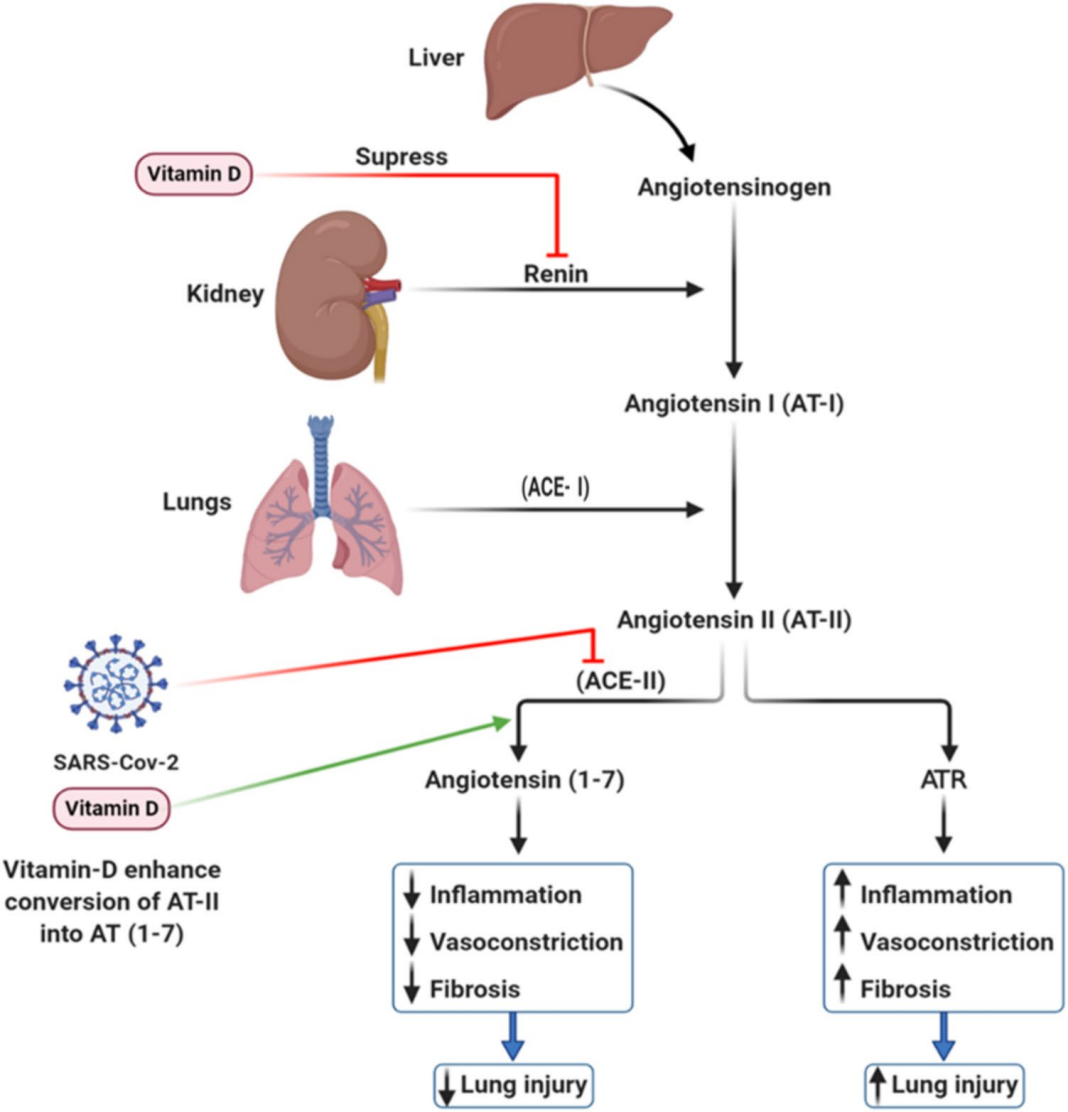
The role of vitamin D in regulating ACE2. The liver produces angiotensinogen converted to angiotensin I by renin (from the kidney). AT-I is converted to AT-II by ACE1 (from the lungs). In the absence of vitamin D, AT-II is converted to ATR, which leads to inflammation, vasoconstriction, and fibrosis of the lungs. SARS-CoV-2 inhibits AT-II and promotes the ATR pathway. Meanwhile, in the presence of vitamin D, AT-II is converted to angiotensin (1–7), which reduces lung inflammation and fibrosis to keep the lungs intact

Vitamin D controls the renin–angiotensin system (RAS) by inhibiting the release of renin from the kidney, thus decreasing the levels of angiotensin I (AT-I) and then AT-II (Las et al. 2020; Xu et al. 2017) (Fig. 3). This inhibition favors the conversion of AT-I into AT (1–7), which inhibits inflammation and increases vasodilation in the lung (Gorman et al. 2017), maintaining the lung cells integrity. Moreover, vitamin D increases the ratio of ACE2 to ACE, which mediates the hydrolysis of angiotensin II and reduces the inflammatory response and lung injury (Rhodes et al. 2021a). Generally, vitamin D deficiency in patients (especially men, as the RAS system is X-linked) increases the activity of RAS. It renders them more susceptible to cytokine storm, one of the hall clinical marks of COVID-19 (Benskin 2020).

Vitamin D is a fat-soluble prohormone, and its deficiency is associated with many health-related issues (Kheiri et al. 2018). VDD can impair mitochondrial functions and trigger oxidative stress and inflammatory responses. Vitamin sufficiency reduces oxidative stress and improves mitochondrial and endocrine functions (Wimalawansa and Vitamin 2019). Vitamin D receptors are present in different tissues, necessitating a deep understanding of its other potential biological functions (Judd and Tangpricha 2009).

## Vitamin D and the immune system

**Fig. 4:**
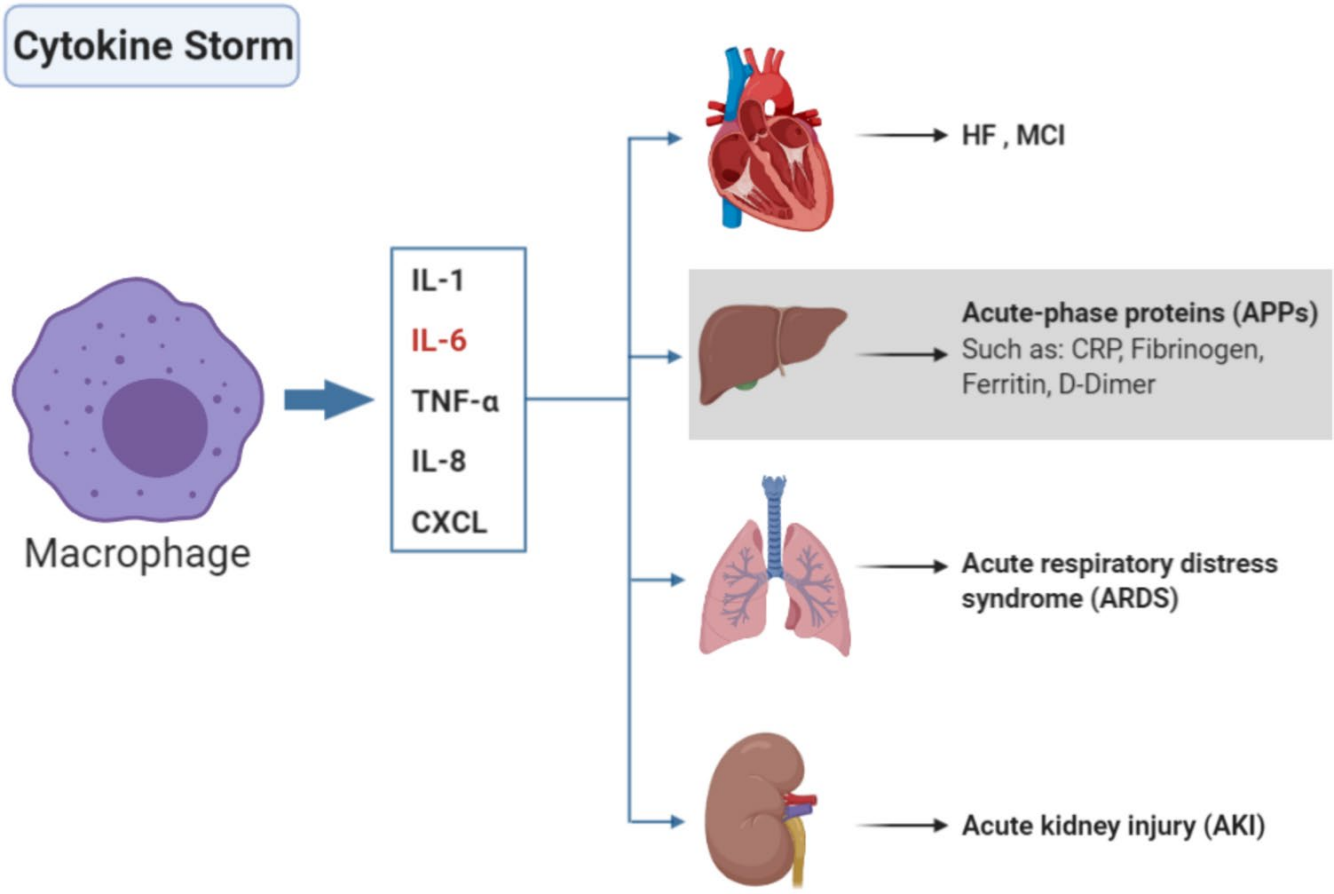
The initiation of the cytokine storm. Without vitamin D, the immune system goes into a bad pathway in which a storm of several cytokines is produced. Macrophages are the main immune cells that produce these cytokines, including IL-1, IL-8, IL-6, TNF-α, and CXCL. This cytokine storm affects several organs, including, but not limited to, the lung, kidney, heart, and liver. The liver accordingly releases CRP, ferritin, D-mimer, and fibrinogen, the main parameters of COVID-19 infections

Vitamin D has demonstrated immune system modulation (Prietl et al. 2013), influencing innate and adaptive immunity (Bilezikian et al. 2020). Research indicates its ability to suppress the synthesis of various cytokines, including IL-6 (Zhang et al. 2012) (Fig. 4). Thus, VDD is associated with the severity of different diseases, including COVID-19 (Kow et al. 2020; Speeckaert and Delanghe 2020). Several reports have indicated that vitamin D deficiency impairs the innate immune system, and the patients who suffer from this deficiency are more vulnerable to COVID-19 (Benskin 2020). Therefore, maintaining an optimal vitamin D level appears crucial for ameliorating the severity of the disease (Yisak et al. 2021).

**Fig. 5:**
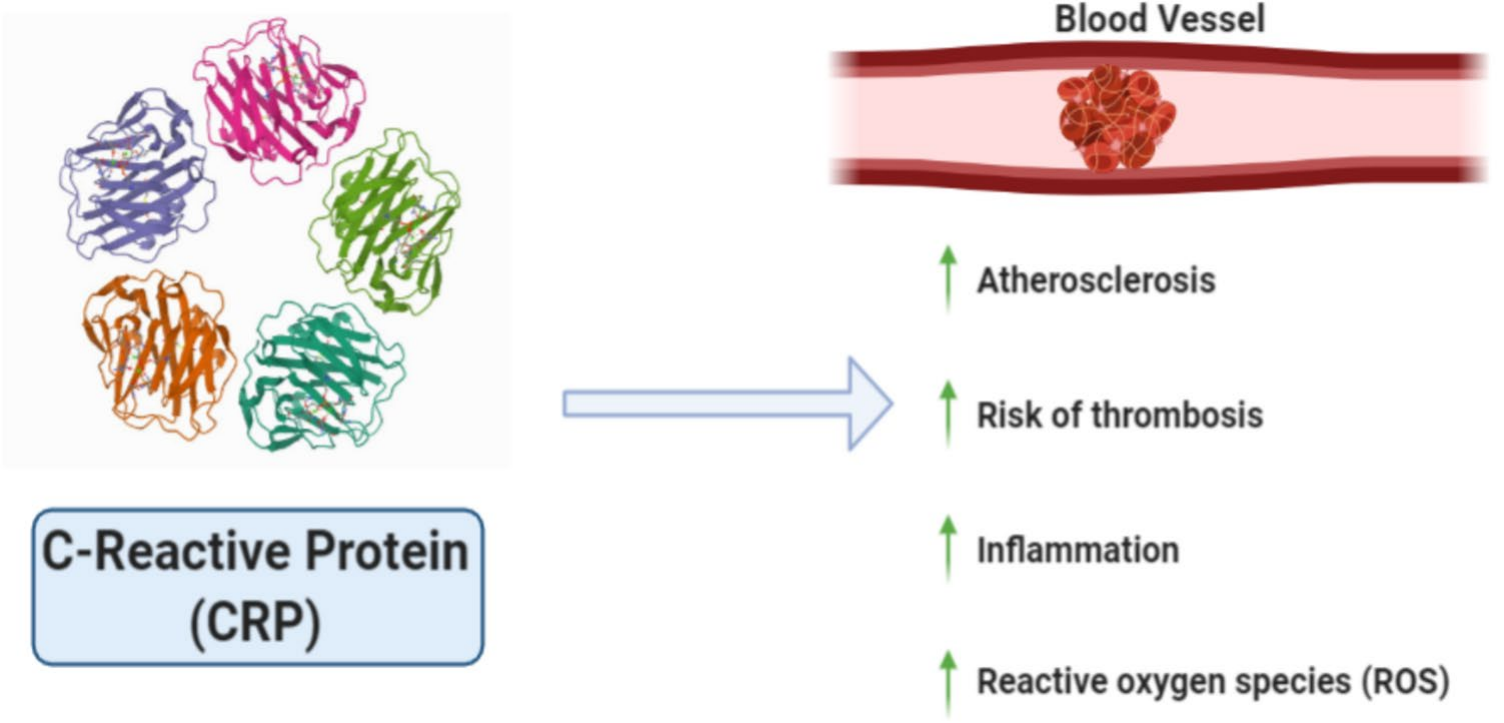
The effect of CRP on blood vessels. When the level of CRP, generated by the liver, increases, it affects the blood vessels through increasing atherosclerosis inflammation and elevated levels of ROS, increasing the likelihood of thrombosis

VDD is associated with an elevation in the level of inflammatory cytokines, which increases the risk of viral upper respiratory tract infections (Barker et al. 2014; Meckel et al. 2016; Weir et al. 2020). Therefore, vitamin D supplementation can reduce the cytokine storm that signifies some of the most serious consequences of COVID-19 (Bilezikian et al. 2020). This catastrophic amount of cytokines released from the liver that includes IL1, IL-8, chemokines, TNF-α, and IL-6 (Ahmed 2020) affects blood vessels by increasing the risk of thrombosis, atherosclerosis, and inflammation, along with the elevated level of ROS (Rhodes et al. 2021b) (Fig. 5).

Furthermore, vitamin D can reduce the risk and seriousness of SARS-CoV-2 infection by blocking viral replication directly (Farid et al. 2021) and promoting the production of cathelicidins (Greulich et al. 2017) and β-defensins (Watts et al. 2020) from macrophages as well as reducing IL-6 production (Miroliaee et al. 2018). These proteins can disrupt the viral phospholipid bilayer and cleave the viral peptide via proteolytic action. Furthermore, cathelicidins trigger vascular permeability, causing the blood vessels to leak out more macrophages at the site of virus accumulation. Generally, these two antimicrobial proteins increase the production of anti-inflammatory cytokines and reduce pro-inflammatory cytokines that induce inflammation and lung injuries, the leading cause of pneumonia (Grant et al. 2020). Vitamin D can also help accelerate the healing process of the affected lung areas (Mohan et al. 2020).

## Immunomodulatory and anti-inflammatory properties of vitamin D in COVID-19

Vitamin D is recognized for its immunomodulatory properties, enhancing innate immune responses while suppressing excessive inflammation and cytokine storms associated with severe COVID-19 (Christakos et al. 2011; Holick 2007). Observational studies demonstrate an association between vitamin D deficiency (< 20 ng mL^−1^) and increased risk of SARS-CoV-2 infection and COVID-19 severity (Hossein-nezhad and Holick 2013). Small clinical trials reveal that high-dose vitamin D3 supplementation (e.g., 60,000 IU weekly) in vitamin D deficient COVID-19 patients rapidly normalizes vitamin D levels and may reduce hospital stay (Manson et al. 2016; Kennel et al. 2010). The mechanisms by which vitamin D may impact COVID-19 pathogenesis include modulating inflammatory cytokine production, stimulating antimicrobial peptides, regulating the renin–angiotensin system, and tempering the NLRP3 inflammasome. NLRP3 is an innate immune receptor that promotes inflammation when excessively activated. By modulating NLRP3, vitamin D may counter the inflammatory pathology in COVID-19 (Christakos et al. 2011; Malabanan et al. 1998). While vitamin D deficiency correlates with worse outcomes, further research is required to establish causality and the therapeutic efficacy of vitamin D supplementation as an adjunctive treatment for COVID-19.

## Vitamin D and its impact on SARS-cov-2 variants

The role of vitamin D in influencing the severity and outcomes of COVID-19 infection has been an area of active investigation. Recent studies have shed light on the potential interactions between vitamin D status and the emergence of SARS-CoV-2 variants. A longitudinal cohort study on the Omicron BA.2 subvariant in children found that those with vitamin D insufficiency had worse clinical outcomes than those with sufficient vitamin D levels (Peng et al. 2022). Mamurova et al. discovered a significant link between variations in the VDR gene and susceptibility to SARS-CoV-2 variants (Mamurova et al. 2023). This connection implies that genetic differences in VDR might contribute to the varying responses seen among different SARS-CoV-2 strains.

In a longitudinal real-world cohort study, Huang et al. focused on vitamin D levels’ impact on COVID-19 pneumonia caused by the Delta variant (Huang et al. 2023). Their findings indicated that individuals deficient in vitamin D tended to experience quicker disease progression, slower recovery, and more pronounced inflammatory markers in their pneumonia compared to those with adequate vitamin D levels. Specifically, those with insufficient vitamin D showed elevated levels of inflammatory markers such as interleukin-6 and procalcitonin, along with delayed viral clearance, as evidenced by higher cycle threshold values for viral gene targets. Likewise, another real-world cohort study focusing on adults infected with the Delta variant showed a correlation between vitamin D deficiency and more severe COVID-19 pneumonia. These observations underscore the potential role of vitamin D in influencing the development and clinical course of COVID-19 pneumonia, particularly in the context of evolving SARS-CoV-2 variants.

Recent evidence suggests that vitamin D may play a crucial role in modulating immune responses and clinical outcomes, particularly with more immune-evasive variants like Omicron and Delta, compared to earlier variants. However, further research is essential to understand the specific mechanisms involved and to determine optimal vitamin D levels for reducing severe outcomes across different lineages of SARS-CoV-2.

## Vitamin D and COVID-19 mortality rate

Recent studies have indicated an inverse association between VDD and an elevated mortality rate in hospitalized COVID-19 patients (Annweiler et al. 2020; Singh et al. 2020a; Panagiotou et al. 2020). Thus, a great interest has recently been raised regarding vitamin D protective and therapeutic role in COVID-19. Reports indicated that very low 25(OH)vitamin D levels (< 10 ng mL^−1^) were highly prevalent and suggestive of deficiency among hospitalized severe COVID-19 patients (Camargo and Martineau 2020). In comparison, low 25(OH) vitamin D levels (< 20–≥ 10 ng mL^−1^) were not associated with the outcome variables (Cereda et al. 2020). Therefore, to decrease the risk of contracting a new infection, it is recommended for people at high risk of COVID-19 to take 10,000 IU d^−1^ of vitamin D for a few weeks, followed by 5000 IU d^−1^ to raise the concentrations of the active form of vitamin D. For those who became infected, elevated doses of vitamin D is recommended (Grant et al. 2020). In addition, vitamin D can protect against ARDS, the main death-causing complication of COVID-19 (Abraham et al. 2021). Moreover, seven out of nine studies reviewed by Yisak et al. (2021) have indicated a crucial role in vitamin D status in COVID-19 infection, prognosis, and mortality. For example, in a study involving 7807 participants, where 782 were COVID‐19 positive and 7025 were COVID‐19 negative, the plasma level of vitamin D was significantly lower in the positive group (19.00 ng mL^−1^) compared with the negative group (20.55 ng mL^−1^) (Merzon et al. 2020).

On the contrary, a retrospective analysis by Mbata et al. found no association between vitamin D deficiency and COVID-19 severity (Mbata et al. 2023). However, the retrospective design limits causal inference, and the study has many limitations, including vitamin D dosage and serum 25-hydroxyvitamin D level measurements that may differ among the included patients. Moreover, Domazet Bugarin et al. conducted a single center underpowered randomized trial of vitamin D supplementation in hospitalized COVID-19 patients, suggesting that there was no benefit in vitamin D supplementation to patients with severe COVID-19 disease admitted to the ICU and in need of respiratory support (Domazet Bugarin et al. 2023). While they found no difference in clinical outcomes, the sample size was small (*n* = 75 per group). This study was underpowered to detect a 2-day difference in respiratory support. The study was originally powered for 137 patients per group. Lastly, Seely et al. performed an underpowered double-blind randomized trial of vitamin C, D, zinc, and vitamin K supplementation versus placebo for 21 days in 90 outpatients with COVID-19 (46 control, 44 treatment) (Dugald et al. 2023). Due to the small sample size, lack of serum 25-hydroxyvitamin D level measurements, and participant-related limitations relevant to this study, the statistical power was significantly reduced. Additionally, due to a lack of recruitment and a low compliance rate, it was difficult to reach a definitive conclusion on the effectiveness of these nutrients.

Nevertheless, the currently available data regarding vitamin D’s protective role is unclear and has conflicting results (Farid et al. 2021; Pereira et al. 2020; Orchard et al. 2021); thus, large,randomized studies are needed to attain a clear conclusion.

## Vulnerable groups

Patients suffering from any form of vitamin D deficiency are at increased risk of getting COVID-19. These groups include the following.

### Elderly people

COVID-19 affects all age groups, with the elderly (older than 65 years of age) being affected more severely (Shahid et al. 2020; Dhama et al. 2020), and they are more susceptible to developing serious clinical symptoms with higher mortality rates (Barros et al. 2021). The obvious higher rates of mortality in the elderly compared with young patients during the COVID-19 pandemic is in part attributed to impairment in the response in older patients (Kadambari et al. 2020; Perrotta et al. 2020). This immune system weakness is partly due to vitamin D deficiency (Benskin 2020; Hribar et al. 2020), and the scenario worsens in the presence of other co-morbidities such as diabetes, hypertension, and cardiovascular diseases, among others (Kumar et al. 2021). Cellular- and immuno-senescence may trigger viral-induced cytokine storm with subsequent life-threatening complications. (Perrotta et al. 2020; Nehme et al. 2020).

Cytokine storm is one of the hallmarks of COVID-19 in older adults, and it happens due to a systemic increase in the pro-inflammatory cytokines. These cytokines induce acute respiratory distress syndrome, pneumonia, and multiple organ failure. It is assumed that a mild to moderate elevation of local and systemic pro-inflammatory cytokines is characterized by inflammaging (Meftahi et al. 2020), the status at which various pro-inflammatory cytokines are produced as an age-associated biological phenomenon. A study by Baktash et al. demonstrated that COVID-19-positive patients have lower levels of serum 25(OH)D level (27 nmol L^−1^) compared with those COVID-19 negative (52 nmol L^−1^) (*p* = 0.0008) (Baktash et al. 2020). Vitamin D has been proposed as a key controller of the inflammatory response, mitochondrial respiration, and ROS production; therefore, it is associated with aging. For example, it reduces the inflammatory response by maintaining optimal levels of Ca^+^ and ROS (Berridge 2017).

### Diabetes

Diabetes mellitus (DM) is one of the most reported co-morbidities associated with the severity of COVID-19 (Singh et al. 2020b). Patients with DM are at increased risk of having severe complications, including acute respiratory distress syndrome (ARDS). It is estimated that 20–50% of COVID-19-positive patients had comorbid diabetes (Bornstein et al. 2020). Furthermore, epidemiological studies indicate that poorly controlled diabetic patients are most likely to be hospitalized because of bacterial, fungal, and/or viral infections (Erener 2020). Thus, a comprehensive understanding of how diabetes deteriorates the prognosis of COVID-19 and how this viral infection also worsens hyperglycemia in diabetic patients is crucial in tailoring customized treatments for better clinical management of these patients (Pal and Bhadada 2020).

Several outcomes characterize DM, such as a weakened immune system, high level of pro-inflammatory cytokine production, and downregulation of ACE2. These features contribute to worsening the prognosis of COVID-19 and predispose patients to severe complications that include coagulopathy, vasculopathy, and psychological stress. On the other hand, cytokine-induced insulin resistance, low levels of calcium, and β-cell damage via dysregulation of ACE2 in the pancreas, which leads to inflammatory responses, are major contributors to the bad prognosis of diabetes. Many clinical studies indicated the association between VDD and COVID-19; a high percentage of these reports showed that VDD is also associated with diabetes (Weir et al. 2020).

Insulin resistance is a hallmark in DM type II, where β-cells secrete more insulin to overcome this resistance and thus prevent the elevation of the blood glucose. Hypersecretion of insulin leads to calcification of B-cells, and ROS signaling results in cell death. VDD has been linked to the onset of DM, contributing to insulin resistance (Berridge 2017; Mezza et al. 2012).

VDD has been proposed to have a role in insulin resistance, where it is associated with polymorphism in different genes, including VDR receptor, vitamin D-binding protein, and vitamin D 1 alpha-hydroxylase. Furthermore, VDD can regulate immune functions via activating innate and adaptive immunity, producing different types of cytokines, activating NFκB, and inducing TNFα. VDD’s effects increase the risk of insulin resistance (Sung et al. 2012).

DNA methylation is a well-known epigenetic mechanism that fundamentally participates in the regulation of gene expression. It is generally accepted that vitamin D can regulate DNA methylation, histone acetylation, and microRNA generation to maintain normal biological functions (Zhou et al. 2015). Several studies have indicated the association between vitamin D and the level of DNA methylation in different genes (Ong et al. 2020). Some diabetes-related genes are epigenetically regulated through hypermethylation of their promoter regions. Vitamin D has been associated with this regulation by preventing hypermethylation via upregulating DMA demethylases, whose function is to prevent the methylation of several diabetes-related genes. Nevertheless, there is a mutuality between the vitamin D system and epigenetic mechanisms, where they regulate each other (Snegarova and Naydenova 2020).

On the other hand, several vitamin D-related genes, such as VDR, CYP2R1, CYP24A1, and CYP27B1, possess CpG stretches in their promoters and hence can be regulated by DNA methylation (Szymczak-Pajor et al. 2020). Furthermore, VDR protein can interact with different chromatin modifiers such as HMTs, HATs, and HDACs. VDR can also target lysine-specific demethylase (LSD) and HDMs of the Jumonji C (JmjC)-domain, given that VDR has DNA demethylating properties (Fetahu et al. 2014).

### Obesity

Obesity triggers chronic inflammation, VDD, and causes mechanical compression of the lungs, which increases the risk of SARS-CoV-2 infection and its complications (Cuschieri and Grech 2020). Since vitamin D is a fat-soluble steroid, the adipose tissue can retain and attract vitamin D, lowering its level in the bloodstream. A low level of the active form of vitamin D leads to the synthesis of more AT-II that binds to the AT receptor. The latter is responsible for lung damage via increasing the inflammatory response and vasoconstriction. Furthermore, the lower vitamin D level directs the differentiation of T Naïve cells into Th1 (the bad pathway), which ultimately results in the cytokine storm, the characterizing phenomenon of COVID-19.

Recent reports have linked VDD with obesity and COVID-19 severity. Evidence indicates a target level of vitamin D of 50 nmol L^−1^, and to reach this level, it is recommended to supplement patients with 800 IU d^−1^ (not 400 IU/day as currently recommended in the UK) (Griffin et al. 2020).

### Ethnicity

As COVID-19 continues its journey across the world, it becomes obvious that its morbidity and mortality rates vary from one nation to the other (Sidiropoulou et al. 2021), indicating that ethnicity might have a role. Considering that skin color—among other factors—can modulate vitamin D levels, melanin is involved in the severity of infection in different populations (Sidiropoulou et al. 2021; Richard et al. 2017). During the COVID-19 pandemic, higher fatality rates were corresponding to VDD rates (Benskin 2020). Earlier reports indicated that Blacks and Hispanics have lower levels of vitamin D compared with Whites (Gutiérrez et al. 2011), and recently, (Mercola et al. 2020) indicated that dark skin color and VDD are features of severe COVID-19 disease. The increased mortality rate among Blacks, who have a reduced UV-B absorption ability, is comparable with the low level of vitamin D. It is now well established that VDD is associated with elevated rates of COVID-19 mortality (Abraham et al. 2021). It has been indicated that African Americans have a 15- to 20-fold higher prevalence of severe VDD than Europeans (Ames et al. 2021). For example, a study involving 110 healthy older African American women found that serum 25-hydroxyvitamin D (25OHD) is lower in women with darker skin color compared with Whites (Gallagher et al. 2013). Another study involving 208 individuals with a mean age of 59.1 years reported that Blacks had lower total 25(OH)D concentrations (20.3 ng mL^−1^) than Whites (26.7 ng mL^−1^) in the USA (Alzaman et al. 2016).

Furthermore, several reports showed that a relatively higher COVID-19 mortality rate is observed in the Northern Hemisphere, with an increase of 4.4% in the mortality for each 1° latitude to the North, considering age. This finding supports the role of UV-B and vitamin D levels (Rhodes et al. 2021a; Chandran et al. 2020).

### Cardiovascular diseases

Several studies have investigated the relationship between vitamin D supplementation and heart failure. It was indicated that vitamin D improved health-related quality of life and CRP levels (Wang et al. 2019). Furthermore, vitamin D decreases serum levels of inflammatory markers in heart failure patients. However, elevated serum vitamin D levels did not reduce cardiac disease-related mortality. Anemia and VDD are common features in chronic heart failure. In a study conducted by Małyszko et al. (2019), 116 CHF and valvular disease patients were enrolled to investigate the levels of vitamin D, VDBP, hemoglobin, and serum creatinine. They found that the prevalence of anemia was 22%, and vitamin D levels were lowest in the valvular disease group. A similar profile has been shown in vitamin D binding protein. Moreover, valvular disease is associated with VDD (Małyszko et al. 2019).

CHF patients suffer from VDD because of their sedentary lifestyle and low outdoor activities, where sunlight is the main source of vitamin D. Furthermore, this deficiency, as indicated by a recent case–control study, is attributed to lack of exposure to UV-B during the early stages of their life (childhood and adolescence) (Zittermann et al. 2006).

## Vitamin D screening and diagnosis recommendations

Screening for vitamin D deficiency is recommended for high-risk individuals, including those with malabsorption disorders, kidney/liver disease, obesity, dark skin pigmentation, and inadequate sun exposure (LeFevre 2015); routine screening is not recommended for healthy adults under 50 years old. However, screening can be considered for those with multiple risk factors (Holick et al. 2011). Older adults (over 65 years) should be screened for VDD due to age-related declines in cutaneous synthesis and intestinal absorption (Kennel et al. 2010).

The diagnosis of vitamin D deficiency, determined by serum 25(OH) vitamin D levels, is considered the most accurate indicator of vitamin D status (Kennel et al. 2010):

1. Levels below 12 ng/mL indicate severe deficiency.
2. Levels between 12 and 20 ng/mL suggest deficiency, with treatment recommended for levels below 30 ng/mL (Holick et al. 2011).
3. The optimal levels are 30–60 ng/mL, with a recommended prophylactic dose (Rosen et al. 2012).

## Prophylaxis of VDD

The recommended daily vitamin D doses vary by age (Manson et al. 2016): (1) Infants (0–1 years): 400 IU/day (10 mcg) of cholecalciferol, equivalent to four drops (400 IU) daily of liquid vitamin D; (2) children (> 1 year): 600 IU (15 mcg) of cholecalciferol, equivalent to six drops (600 IU) daily; (3) children (> 8 years) half an adult dose of vitamin D drops daily, and (4) adults: the recommended prophylactic dose is either one full adult dose (600–800 IU/day) of vitamin D drops or 2000 IU of cholecalciferol in tablet form daily (Manson et al. 2016; Malabanan et al. 1998; Ish-Shalom et al. 2008).

## Treatment of VDD

Individuals with serum 25-hydroxyvitamin D levels below 30 ng mL^−1^ are considered vitamin D deficient and warrant treatment (Kennel et al. 2010). A treatment dose of 50,000 IU of vitamin D is recommended weekly. The treatment can be administered via various routes and regimens:

1. Oral cholecalciferol (vitamin D3): 50,000 IU once weekly for 8 weeks (Malabanan et al. 1998).
2. Ergocalciferol (vitamin D2): 50,000 IU once weekly for 8 weeks (Malabanan et al. 1998).
3. Injectable cholecalciferol: 200,000 IU administered intramuscularly or subcutaneously once a month for 2 months (Ish-Shalom et al. 2008).

Concomitant calcium supplementation is recommended to enhance the effects of vitamin D therapy (Tang et al. 2007).

After achieving adequate vitamin D levels, patients should continue with maintenance therapy. Maintenance doses are typically lower than treatment doses, ranging from 800 to 2000 IU/day for adults (Institute of Medicine Committee to Review Dietary Reference Intakes for Vitamin D, Calcium 2011). Regular sun exposure and dietary vitamin D intake also help to maintain vitamin D levels within the normal range.

Vitamin D toxicity is rare, but can occur from extremely high doses over long periods. Symptoms include nausea, vomiting, poor appetite, constipation, weakness, and kidney problems (Hathcock et al. 2007). Serum 25(OH)vitamin D levels consistently over 150 ng mL^−1^ indicate toxicity (Jones 2008). Routine toxicity monitoring is unnecessary for doses under 10,000 IU/day (Vieth 1999).

Patient education on treatment duration and compliance is crucial, with an emphasis on adhering to the prescribed treatment regimen for a duration of 2 months. Afterward, a transition to maintenance prophylactic doses (e.g., 1000 IU vitamin D tablet daily) is advised.

Vitamin D is a fat-soluble vitamin; therefore, it is advisable to take supplements with a meal rich in fat to optimize absorption (Mulligan and Licata 2010).Enough sun exposure is recommended to avoid potential adverse effects, and tanning beds should be limited (Gambichler et al. 2002). It is recommended to have serum vitamin D levels reassessed within 3–4 months after initiating treatment (Kennel et al. 2010).

Various medications can influence vitamin D levels. For example, antiepileptic drugs like phenytoin and carbamazepine may accelerate vitamin D metabolism, leading to lower serum levels. Glucocorticoids can impair vitamin D metabolism and decrease calcium absorption, thus adversely affecting vitamin D Levels in serum. Additionally, drugs that alter gastrointestinal absorption, such as orlistat, may reduce vitamin D absorption in diet from the intestines. Moreover, the oral solution should be stored at a temperature not exceeding 30 °C and must be protected from exposure to light to ensure its stability and effectiveness (Brown and Josse 2002).

## Molnupiravir and paxlovid as adjunct for COVID-19 teatment

Molnupiravir is the orally bioavailable prodrug of N4-hydroxycytidine, a ribonucleoside analog that induces viral mutagenesis. It is metabolized into the active N4-hydroxycytidine triphosphate form, which competes with natural cytidine during SARS-CoV-2 replication, causing mutations in the viral genome that accumulate to the point of viral suppression (Niraj et al. 2022). Molnupiravir was granted Emergency Use Authorization (EUA) by the US Food and Drug Administration (FDA) in December 2021 for the treatment of mild-to-moderate COVID-19 in high-risk adults (FDA 2021a). The drug is developed and marketed by Merck & Co.

Paxlovid is an oral antiviral combining nirmatrelvir, a 3CL protease inhibitor, with ritonavir, a CYP3A inhibitor for pharmacokinetic boosting. By blocking the 3CL protease enzyme essential for viral replication, nirmatrelvir suppresses SARS-CoV-2 propagation (Owen et al. 2021). Paxlovid obtained EUA from the FDA in December 2021 and is authorized for the treatment of mild–moderate COVID-19 in high-risk patients aged ≥ 12 years and weighing ≥ 40kg (FDA 2021b). Pfizer and BioNTech jointly developed it.

Vitamin D is known for its immunomodulatory effects, including the enhancement of innate antiviral immunity and the modulation of inflammation (Arboleda and Urcuqui-Inchima 2020). Observational studies have linked vitamin D deficiency to an increased susceptibility to respiratory viral infections (Martineau et al. 2019). Though not a direct antiviral itself, vitamin D may provide synergistic benefits when combined with direct-acting antivirals by optimizing the immune response against SARS-CoV-2. However, clinical assessment is required to evaluate vitamin D as an immunomodulatory adjunct to molnupiravir and paxlovid for COVID-19 treatment.

### Investigating vitamin D treatment for COVID-19

**Table:** 

Type of study	Author/country	Sample size	Intervention	Outcomes	Refs
Randomized controlled trial	Murai et al. (Brazil)	240 patients	200,000 IU vitamin D3 (single dose) or placebo	No significant difference in length of stay	Murai et al. 2020)
Randomized controlled trial	Entrenas Castillo et al. (Spain)	76 patients	532 mg Calcifediol (25(OH)D3) on day 1, 0.266 mg on days 3 and 7, then weekly	Reduced ICU admissions	Castillo et al. 2020)
Randomized controlled trial	Sabico et al. (Saudi Arabia)	348 patients	5000 IU D3 daily or placebo for 2 weeks	No difference in disease severity or mortality	Sabico et al. 2021)
Randomized controlled trial	Rastogi et al. (India)	76 patients	High-dose vitamin D (60,000 IU daily for days)	Improved inflammatory markers	Rastogi et al. 2022)
Randomized controlled trial	Lakkireddy et al. (India)	40 patients	60,000 IU vitamin D3 or placebo weekly	Improved inflammatory markers	Lakkireddy et al. 2021)
Prospective randomized controlled trial	Sarhan et al. (Egypt)	116 patients with verified COVID-19 hyperinflammation status	Oral alfacalcidol (1 mcg/day) as a vitamin D supplement and intramuscular cholecalciferol (200,000 IU) as a high-dose	The high-dose vitamin D group showed shorter hospital stays, reduced need for oxygen therapy and mechanical ventilation, higher clinical improvement rates, lower sepsis incidence, and significant improvements in laboratory parameters	Sarhan et al. 1358)
Randomized controlled trial	Bugarin et al. (Croatia)	155 subjects (78 in the intervention group, 77 in the control group)	Vitamin D supplementation during their intensive care unit (ICU)	No statistically significant difference in the main outcome of the number of days spent on respiratory support	Domazet Bugarin et al. 2023)
Randomized controlled trial	Jaun et al. (Switzerland)	211 hospitalized COVID-19 patients with vitamin D deficiency	Single high-dose vitamin D substitution (140,000 IU)	Significant reduction in length of hospital stay	Jaun et al. 2023)
Prospective, open-label, RCT	Dilokpattanamongkol et al. (Thailand)	692 COVID-19 pneumonia patients	Oral alfacalcidol (2 mcg daily or < 0.05 mcg/kg/day) until the end of hospitalization	Potential benefits in reducing pneumonia severity and treatment duration	Dilokpattanamongkol et al. 2024)
Pilot randomized trial	Maghbooli et al. (Iran)	66 patients	Calcifediol or placebo	Reduced ICU admission with treatment	Maghbooli et al. 2021)
Open-label trial	Annweiler et al. (France)	76 patients	High-dose vitamin D3 (80,000 IU single dose) or no intervention	Reduced risk of mortality	Annweiler et al. 2022)
Prospective cohort	Ye et al. (UK)	443 patients	Baseline vitamin D levels	Lower mortality with sufficient levels	Ye et al. 2021)
Prospective cohort	Jain et al. (India)	1064 patients	Baseline vitamin D levels	Higher mortality with deficiency	Jain et al. 2020)
Retrospective cohort	Lau et al. (USA)	4638 patients	Prior vitamin D levels	Lower positivity with higher levels	Lau et al. 2020)
Retrospective cohort	Kaufman et al. (USA)	489 patients	Prior vitamin D levels	Higher mortality with deficiency	Kaufman et al. 2020)
Retrospective cohort	Karahan & Katırcı (Turkey)	149 patients	Pre-illness vitamin D levels	Increased mortality with deficiency	Karahan and Katkat 2021)
Retrospective study	De Smet et al. (Belgium)	186 patients	Prior vitamin D supplementation	Lower COVID-19 mortality with supplement	Smet et al. 2021)
Case–control study	Tan et al. (Singapore)	622 cases, 3109 controls	Prior vitamin D levels	Lower COVID-19 odds with higher levels	Tan et al. 2020)
Case–cControl study	Vassiliou et al. (Greece)	134 cases, 194 controls	Baseline vitamin D levels	Lower positivity with higher levels	Vassiliou et al. 2020)
Case–control study	Abdollahi et al. (Iran)	109 cases, 109 controls	Baseline vitamin D levels	Lower positivity with sufficient levels	Abdollahi et al. 2021)
Case–control study	Ahmed et al. (Egypt)	80 subjects (40 COVID-19 patients and 40 healthy controls)	Measurement of serum 25 (OH) vitamin D levels by ELISA technique	Vitamin D insufficiency is associated with higher C-reactive protein, higher D-dimer levels, higher interleukin-6 (IL-6) levels, and higher need for assisted ventilation	Ahmed et al. 2020)

### Bibliometric analysis of vitamin D and immunity in COVID-19 research

**Fig. 6:**
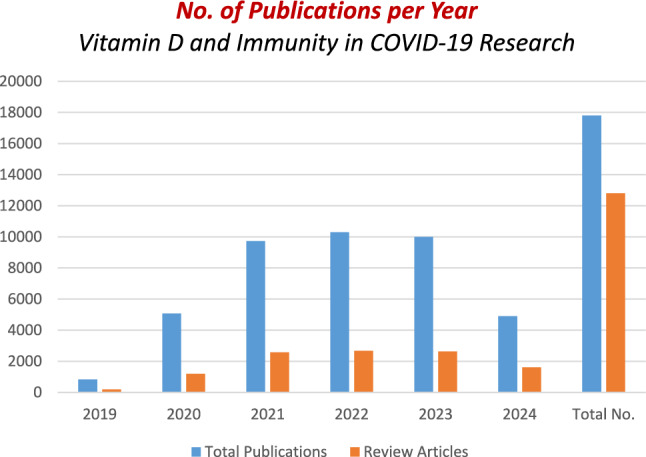
Trends in publications on vitamin D and immunity in COVID-19 research from 2019 to 2024. This figure displays the number of publications related to (“vitamin D” or “cholecalciferol”) and (“COVID-19” or “SARS-CoV-2”) and (immunity or immune) over the years. The data, sourced from Google Scholar (www.googlescholar.com, accessed August 27, 2024), includes a total of 17,800 publications

In the rapidly evolving field of COVID-19 research, bibliometric analysis has become an invaluable tool for mapping and classifying key aspects of scientific literature. This analysis is particularly useful for examining the complex relationships between vitamin D, immunity, and COVID-19. To provide a comprehensive overview, we conducted a bibliometric analysis using the Google Scholar platform, focusing on publications related to (“vitamin D” or “cholecalciferol”) and (“COVID-19” or “SARS-CoV-2”) and (immunity or immune). Figure 6 presents a profile of the current status of studies in this field. The data indicates a steady increase in publications related to these search terms over the years, reflecting a growing interest and activity in this research area. The analysis encompasses annual cumulative publications, including research articles, reviews, editorial material, and letters.

Notably, there has been a significant rise in cited research articles, suggesting that the understanding of the impact of vitamin D on immunity is increasing. This increase supports the ongoing exploration of vitamin D as a potential tool for managing infectious diseases, particularly COVID-19.

The high proportion of review articles—approximately 12,800 out of 17,800 publications—indicates a substantial effort by researchers to synthesize and analyze existing knowledge on the interplay between vitamin D, immunity, and COVID-19. The publications peaked in 2022, followed by a slight decline in 2023 and 2024. Moreover, Fig. 6 outlines the distribution of research and review articles central to this field. This visual representation helps understand the trends and focus areas within the research landscape.

Initially, Google Scholar was used to get a broad overview and estimate the total volume of research. Then, the Web of Science was used for detailed bibliometric analysis, providing precise and curated data essential for in-depth research insights. This approach combines a broad initial view with detailed analysis, similar to methods used in related studies (Tony and Nabwey 2024). A bibliometric analysis was performed using the Web of Science core collection to provide a comprehensive overview of the research on vitamin D and immunity in the context of COVID-19. The search items were (“vitamin D” or “cholecalciferol”) and (“COVID-19” or “SARS-CoV-2”) and (immunity or immune). Subsequently, the data revealed from the “Web of Science Core Collection” database and the VOS viewer software (version 1.6.20) were extracted from 2019 to August 2024. Data was exported in BibTeX format, including titles, abstracts, keywords, authors, publication years, journal names, and citation counts. This data was analyzed using VOS viewer software, which employs co-occurrence analysis, clustering, and network visualization algorithms. Key features include overlay and density visualizations representing term relationships and research focus areas. The purpose of the design is to analyze the keywords of the research articles. The designed mapping incorporates a network. Then, the overlay and density visualization mapping are designed using this software. The current search is based on the co-occurrence associated with authors’ keywords and is designated the minimum occurrence number.

**Fig. 7:**
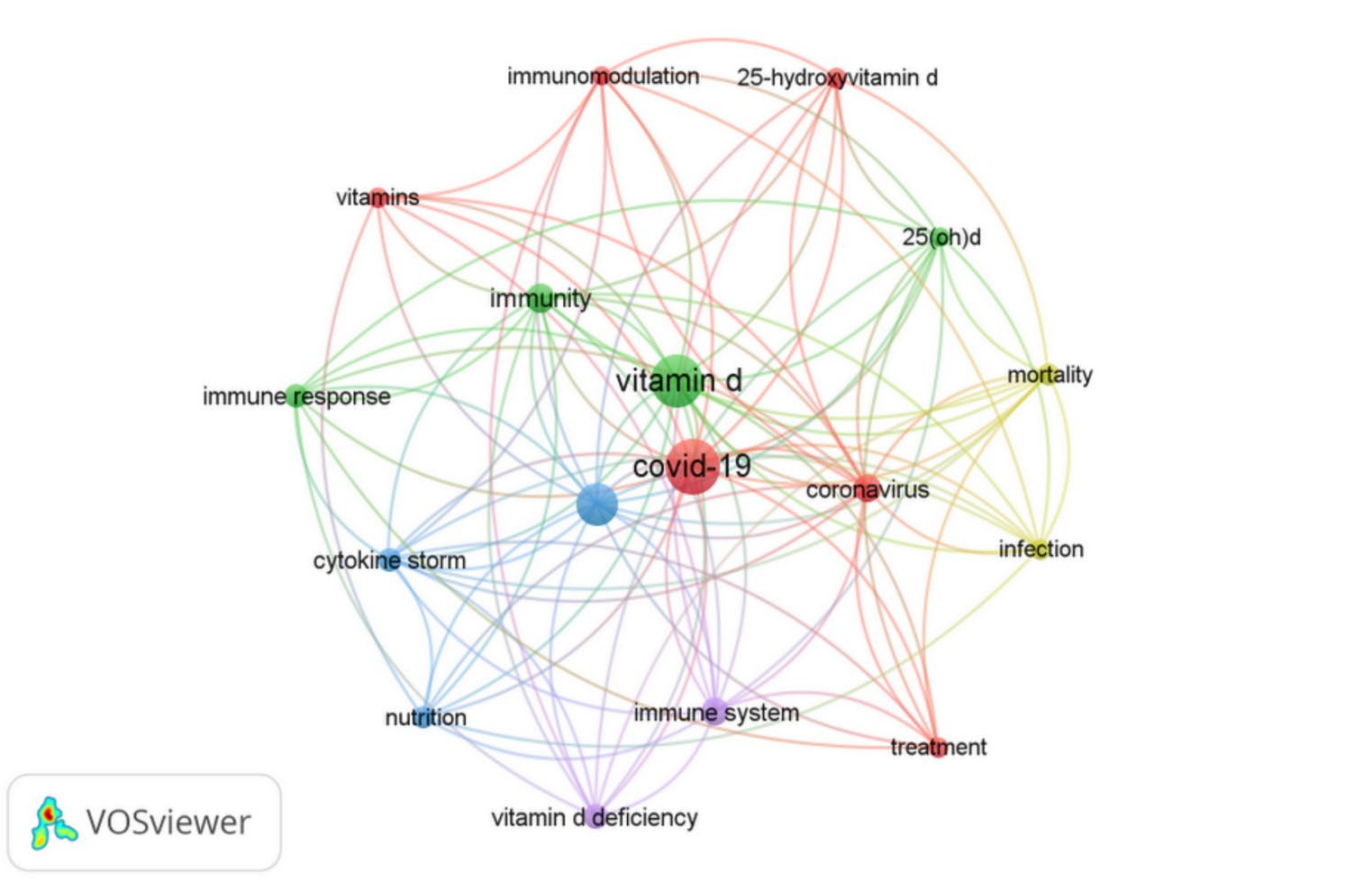
Network visualization of key terms in vitamin D and immunity research related to COVID-19**.** This map, generated using a VOS viewer, illustrates the co-occurrence network of keywords from the Web of Science Core Collection publications. The visualization reveals clusters of related research topics and highlights key areas of study

Consequently, the software adjusts the parameters, and the mapping is designed. The most common feature of the VOS viewer is overlay visualizations, which are used to categorize density visualization over periods. The cited research articles on vitamin D and immunity in COVID-19 research showed that the bibliometric mapping of clusters could be identified as seen in Fig. 7**.** The hotspot clusters can demonstrate the intensive research studies based on the analyzed results attained via the data from “Web of Science” core collection from the search terms (“vitamin D” or “cholecalciferol”) and (“COVID-19” or “SARS-CoV-2”) and (immunity OR immune).

**Fig. 8:**
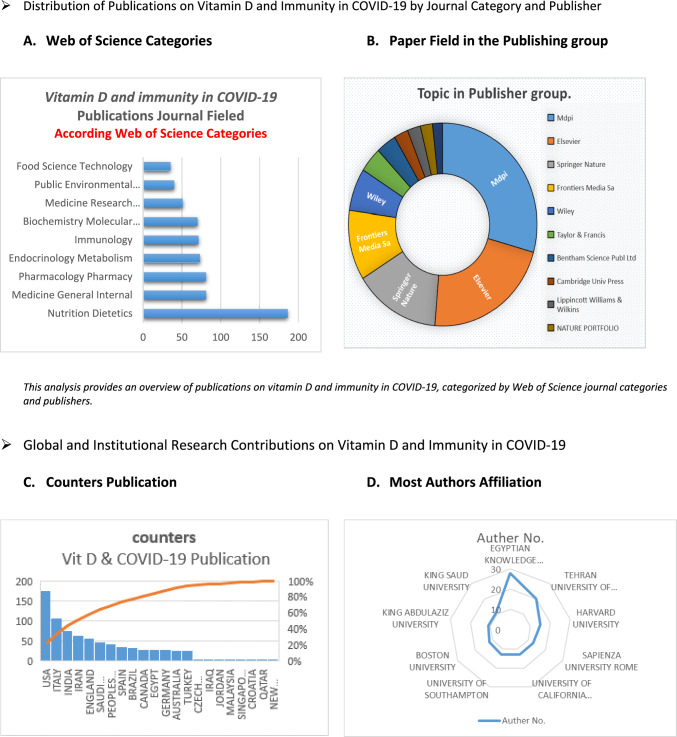
A foundational overview of the research landscape on vitamin D and immunity in COVID-19, based on Web of Science data. A Publications are predominantly in Nutrition Dietetics (186 papers) and Medicine General Internal (81 papers), with major contributions from MDPI (169 papers), Elsevier (126 papers), and Springer Nature (85 papers), highlighting the key role of nutrition and general medicine. B The primary focus is on Nutrition, Dietetics, and Medicine in General Internal, with additional emphasis on Pharmacology, Pharmacy, and Endocrinology Metabolism. C reveals the leading countries in this research are the USA (175 publications), Italy (106), and India (76), with significant input from Iran, England, and Saudi Arabia. D Major institutional affiliations, including the Egyptian Knowledge Bank EKB (28 papers), Tehran University of Medical Sciences (20 papers), and Harvard University (15 papers)

Descriptive data on journal categories (e.g., Nutrition, Medicine), major publishers (e.g., MDPI, Elsevier), leading countries (e.g., USA, Italy), and key institutional affiliations (e.g., Egyptian Knowledge Bank, Harvard University. Figure 8 provides a foundational overview of the research landscape on vitamin D and immunity in COVID-19, based on Web of Science data. This preliminary information highlights key areas of focus, major contributors, and publication trends, setting the context for more detailed bibliometric analysis. By establishing these broad patterns, the study can better interpret and contextualize the network visualizations produced by VOSviewer. The VOSviewer figures will then offer insights into the relationships and collaborations within the research community, allowing for a more nuanced understanding of the field’s development and connections.

This research landscape on vitamin D and immunity in COVID-19 through a detailed analysis of Web of Science data. Publications are predominantly concentrated in Nutrition, Dietetics, and Medicine General Internal, with significant contributions from MDPI, Elsevier, and Springer Nature. Leading countries in this research are the USA (175 publications), Italy (106), and India (76). Key institutions include the Egyptian Knowledge Bank EKB (28 papers), Tehran University of Medical Sciences (20 papers), and Harvard University (15 papers). This descriptive data sets the stage for the subsequent bibliometric analysis using VOSviewer, which will offer a network visualization of research trends and collaborations.

### Analysis of authors and research collaborations

**Fig. 9:**
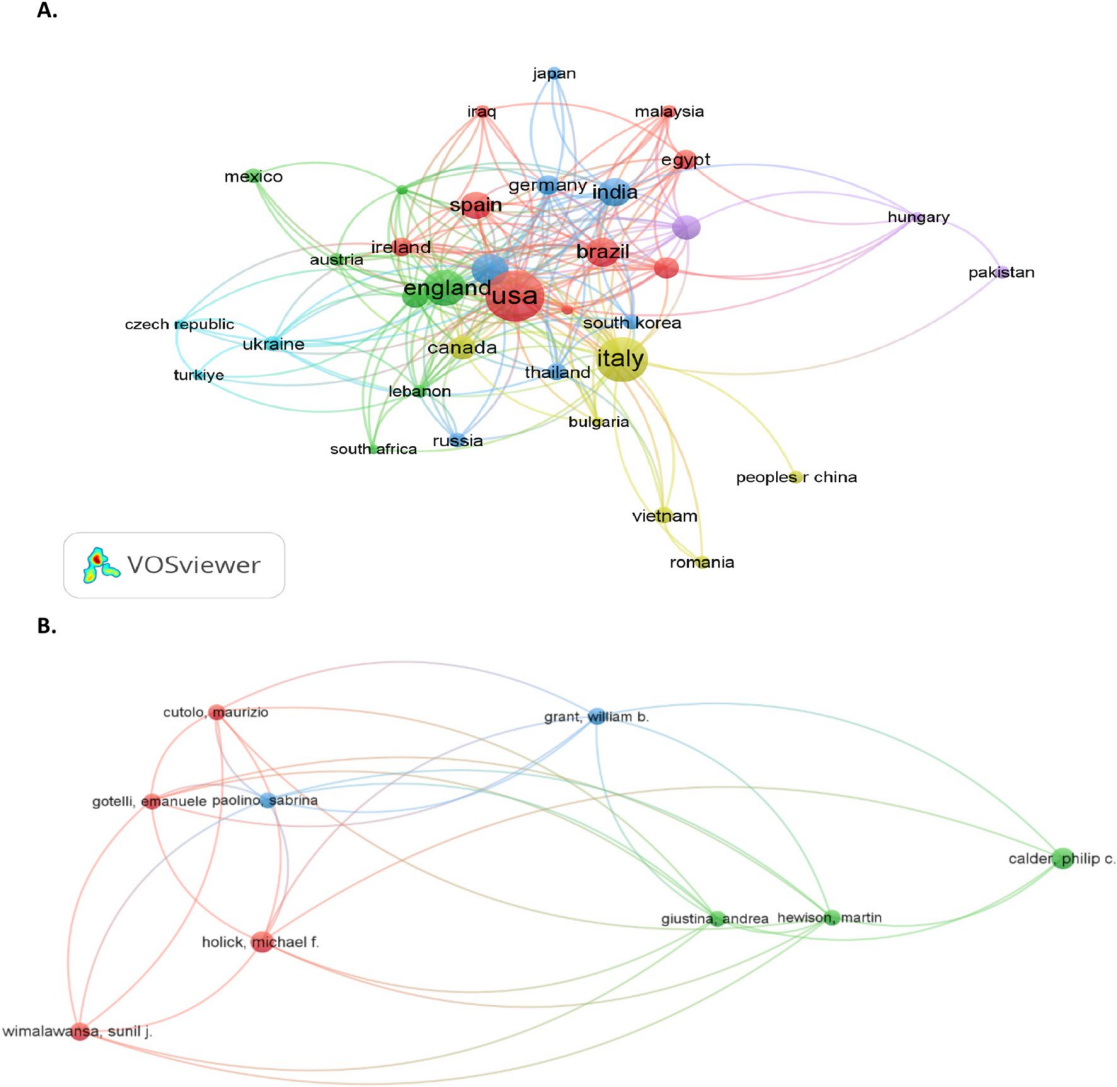
Bibliometric network mapping generated via VOS viewer. **A** Network map showing the collaborations between various countries in the use of vitamin D in COVID-19; **B** co-authorship overlap visualization map for vitamin D and immunity in COVID-19

To get a superior signification of the frontiers in vitamin D and immunity in COVID-19, a bibliometric analysis for the leading researchers and authors or team works for the most creative scientists in the field is conducted using VOS viewer. The most active and productive countries in terms of vitamin D and immunity to COVID-19 are the USA, Italy, England, India, and Egypt, as displayed in Fig. 9A. The item’s weight signifies the size of the cluster and the label.

Hence, the more superior the country’s weight, the greater are ithe item’s label and circle, as shown in Fig. 9A. The information in Fig. 9B. follows the research papers by the main authors. The mapping signifies the authors with the most strength are Holick, MF, Grant, WB, Wimalawansa, SJ, Giustina, A, and their co-workers, who mainly focused on vitamin D and immunity in COVID-19. Notably, there is a shortage in the cooperation of researchers from different organizations and countries that require prospective work. Additionally, there is still a gap in the work done in the field and a conclusive result.

**Fig. 10:**
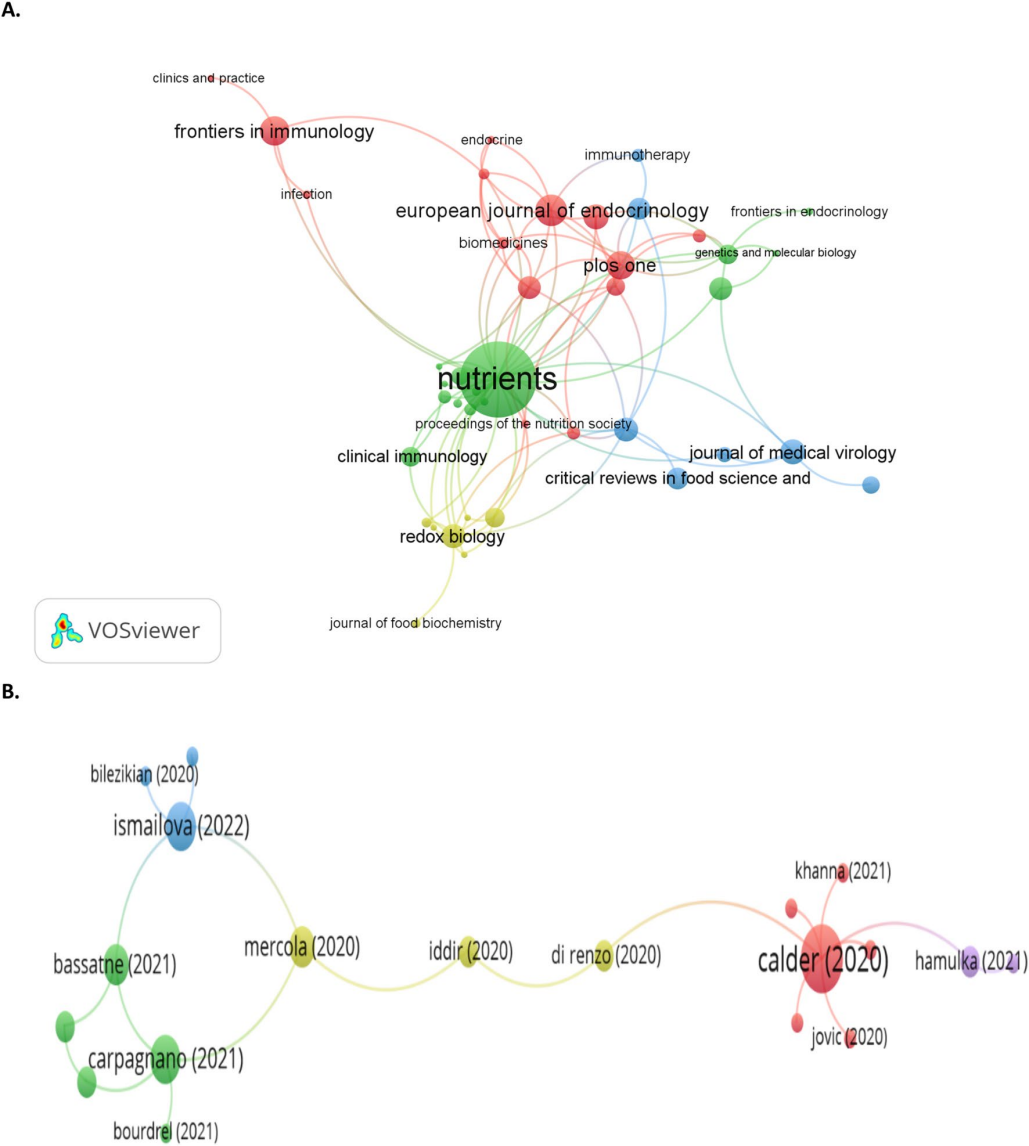
Article sources **A** and citation authors **B** network visualization map in vitamin D and immunity in COVID-19

Bibliometric analysis is a promising technique that could reveal the internal links of published documents (as displayed in Fig. 10. A). It was found that most articles were cited in “Nutrients”, “Metabolism-Clinical and Experimental”, “European Journal of Endocrinology”, “Nutrition Reviews”, “International Journal of Obesity”, and “PLoS ONE”. It is noteworthy that researchers appear to categorize published articles in high-quality journals.

Furthermore, the citation article visualization report is considered and displayed in Fig. 10 B. As can be seen from the mapping, Iddir et al. (2020), Mercola et al. (2020), Bassatne (2021), and Ismailova and White (2022) have the greatest clusters, which signifies such authors possess the most cited articles in the field.

Moreover, Calder et al. (2020) and Carpagnano et al. (2021) are also signified as the most cited articles. Thus, the author’s collaboration analysis indicates that the groups are typically continuous research groups. Those authors study vitamin D and immunity in COVID-19. They cited a continued direction of investigation till now, while the number of publications is also considerable compared to other research groups. However, it is noteworthy to mention that the cooperation of scientists between various institutions and countries is required to be further enhanced (Calder et al. 2020; Carpagnano et al. 2021).

## Conclusion

Despite the huge number of publications, COVID-19 still has many obscure issues that require intensive investigation. Correction of VDD with high-dose supplements in infected patients may provide synergistic benefits with antiviral therapy, leading to improved outcomes. Reviewing the influence of VDD on COVID-19 pathophysiology and vitamin D’s molecular mechanisms would raise awareness and inform strategies to protect people from developing severe complications in case of future pandemics. Well-designed randomized controlled trials are still needed to understand better if VDD is a modifiable risk factor for COVID-19 outcomes. Randomized controlled trials are also needed to establish vitamin D as an adjuvant to antivirals for COVID-19 prevention and treatment. The available mechanistic and epidemiological data suggest that maintaining adequate vitamin D levels may potentially benefit the prevention and treatment of COVID-19, but supplementation cannot yet be recommended based on current evidence alone. Monitoring population vitamin D levels and correcting deficiency remain prudent public health strategies at this time.

Bibliometric analysis highlights the rapid growth in research examining vitamin D’s multifaceted roles in COVID-19. However, despite the increasing volume of studies, there remains a need for large, well-designed, randomized controlled trials to conclusively establish the efficacy and optimal dosing of vitamin D supplementation in preventing and mitigating acute and long-term complications of COVID-19.

## Data Availability

Not applicable.

## Abbreviations

ARDS: Acute respiratory distress syndrome
UV-B: Ultraviolet-B
VDD: Vitamin D deficiency
VDR: Vitamin D receptor
RAS: Renin–angiotensin system
AT-I: Angiotensin I
AT-II: Angiotensin II
ACE: Angiotensin-converting enzyme
CXCL: Chemokine (C-X-C motif) ligand 1 (CXCL1)
IL-6: Interleukin 6
IL-1: Interleukin 1
IL-8: Interleukin 8
TNF-α: Tumor necrosis factor-alpha
ROS: Reactive oxygen species
DM: Diabetes mellitus
NFκB: Nuclear factor kappa-B
DMA: Dimethylaniline
CYP: Cytochrome P
HMTs: Histone methyltransferases
HATs: Histone acetyltransferases
HDACs: Histone deacetylases
LSD: Lysine-specific demethylase
HDMs: Histone demethylases
CRP: C-reactive protein
CHF: Chronic heart failure
VDBP: Vitamin D-binding protein
NLRP3: Nod-like receptor protein 3
