# Correction: Vitamin D: a key player in COVID‑19 immunity and lessons from the pandemic to combat immune‑evasive variants

**DOI:** 10.1007/s10787-025-01725-x

**Published:** 2025-04-12

**Authors:** Hussein Sabit, Shaimaa Abdel-Ghany, Mahmoud S. Abdallah, Osama Abul-Maaty, Ahmed I. Khoder, Nabil A. Shoman, Mohamed Sameh Farrag, Pavel Martasek, Ayman M. Noreddin, Mahmoud Nazih

**Affiliations:** 1 Department of Medical Biotechnology, College of Biotechnology, Misr University for Science and Technology, P. O. Box 77, Giza, Egypt; 2 Department of Environmental Biotechnology, College of Biotechnology, Misr University for Science and Technology, P. O. Box 77, Giza, Egypt; 3 Department of Clinical Pharmacy, Faculty of Pharmacy, University of Sadat City (USC), Sadat City, 32897 Egypt; 4 Department of PharmD, Faculty of Pharmacy, Jadara University, Irbid, 21110 Jordan; 5 Faculty of Science, Mansoura University, Mansoura, Egypt; 6 Scientific Office, Egyptian Society of Pharmacogenomics and Personalized Medicine (ESPM), Cairo, Egypt; 7 Molecular Biology Department, Genetic Engineering and Biotechnology Research Institute, University of Sadat City, Sadat City, Egypt; 8 Pharmacology and Toxicology Department, Faculty of Pharmacy, Menoufia University, Shebin El-Koum, Egypt; 9 Department of Pharmaceutics and Pharmaceutical Technology, Faculty of Pharmacy, Ahram Canadian University, Giza, Egypt; 10 Department of Paediatrics and Inherited Metabolic Disorders, First Faculty of Medicine, Charles University and General University Hospital in Prague Ke Karlovu 2, 128 08 Praha 2, Czech Republic; 11 Department of Clinical Pharmacy, Faculty of Pharmacy, Ahram Canadian University (ACU), 6th of October City, Giza, 12566 Egypt; 12 Department of Internal Medicine, School of Medicine, University of California-Irvine, Irvine, CA USA; 13 Al Ryada University for Science and Technology (RST), ElMehwar ElMarkazy-2, Cairo - Alex desert RD K92, Sadat City, 16504 Egypt

**Correction: Inflammopharmacology (2024) 32:3631–3652** 10.1007/s10787-024-01578-w

In this article, the volume, issue and page number were missing for reference Barassi et al. The incorrect and correct versions are given below.

Incorrect version:

The paper is cited as: Barassi A, Pezzilli R, Mondoni M, Rinaldo RF, DavÌ M, Cozzolino M et al (2021) Vitamin D in severe acute respiratory syndrome coronavirus 2 (SARS-CoV-2) patients with non-invasive ventilation support. Panminerva Med. 10.23736/S0031-0808.21.04277-4

The correct version:

Barassi A, Pezzilli R, Mondoni M, Rinaldo RF, Davi M, Cozzolino M et al (2023) Vitamin D in SARS-CoV-2 patients with non-invasive ventilation support. Panminerva Med **65(1):23–29.** 10.23736/S0031-808.21.04277-4

The original article has been corrected.

